# Crystal structure of 3-[(4-benzyl­piperazin-1-yl)meth­yl]-5-(thio­phen-2-yl)-2,3-di­hydro-1,3,4-oxa­diazole-2-thione

**DOI:** 10.1107/S2056989015002273

**Published:** 2015-02-13

**Authors:** Fatmah A. M. Al-Omary, Ali A. El-Emam, Hazem A. Ghabbour, C. S. Chidan Kumar, Ching Kheng Quah, Hoong-Kun Fun

**Affiliations:** a Department of Pharmaceutical Chemistry, College of Pharmacy, King Saud University, PO Box 2457, Riaydh 11451, Saudi Arabia; b King Abdullah Institute for Nanotechnology (KAIN), King Saud University, Riyadh 11451, Saudi Arabia; c Department of Pharmaceutical Chemistry, College of Pharmacy, King Saud University, Riyadh 11451, Saudi Arabia; d X-ray Crystallography Unit, School of Physics, Universiti Sains Malaysia, 11800 USM, Penang, Malaysia; e Department of Chemistry, Alva’s Institute of Engineering & Technology, Mijar, Moodbidri 574 225, Karnataka, India

**Keywords:** crystal structure, 1,3,4-oxa­diazole, piperazin-1-yl, disorder, π–π inter­actions, S⋯S contacts

## Abstract

The title 1,3,4-oxa­diazole-2-thione derivative, C_18_H_20_N_4_OS_2_, crystallized with two independent mol­ecules (*A* and *B*) in the asymmetric unit. The 2-thienyl rings in both mol­ecules are rotationally disordered over two orientations by approximately 180° about the single C—C bond that connects it to the oxa­diazole thione ring; the ratios of site occupancies for the major and minor components were fixed in the structure refinement at 0.8:0.2 and 0.9:0.1 in mol­ecules *A* and *B*, respectively. The 1,3,4-oxa­diazole-2-thione ring forms dihedral angles of 7.71 (16), 10.0 (11) and 77.50 (12)° (mol­ecule *A*), and 6.5 (3), 6.0 (9) and 55.30 (12)° (mol­ecule *B*) with the major and minor parts of the disordered thio­phene ring and the mean plane of the adjacent piperazine ring, respectively, resulting in approximately V-shaped conformations for the mol­ecules. The piperazine ring in both mol­ecules adopts a chair conformation. The terminal benzene ring is inclined towards the mean plane of the piperazine ring with N—C—C—C torsion angles of −58.2 (3) and −66.2 (3)° in mol­ecules *A* and *B*, respectively. In the crystal, no inter­molecular hydrogen bonds are observed. The crystal packing features short S⋯S contacts [3.4792 (9) Å] and π–π inter­actions [3.661 (3), 3.664 (11) and 3.5727 (10) Å], producing a three-dimensional network.

## Related literature   

For the biological activity of 1,3,4-oxa­diazole derivatives, see: Al-Deeb *et al.* (2006 ▸); El-Emam *et al.* (2004 ▸); Kadi *et al.* (2007 ▸); Padmavathi *et al.* (2009 ▸). For the synthesis of the title compound, see: Al-Omar (2010 ▸). For related 1,3,4-oxa­diazole structures, see: El-Emam *et al.* (2012 ▸, 2013 ▸).
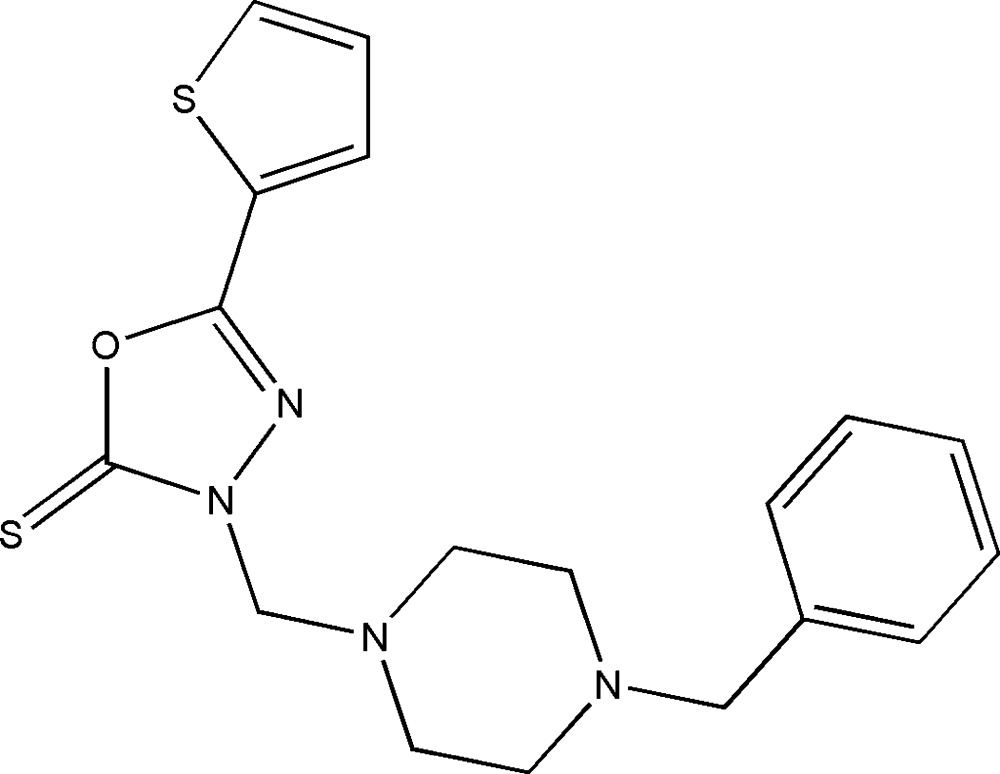


## Experimental   

### Crystal data  

C_18_H_20_N_4_OS_2_
*M* *_r_* = 372.50
Monoclinic, 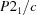

*a* = 10.6909 (5) Å
*b* = 29.3658 (13) Å
*c* = 15.6179 (6) Å
β = 130.283 (2)°
*V* = 3740.4 (3) Å^3^
*Z* = 8
Mo *K*α radiation
μ = 0.30 mm^−1^
*T* = 273 K
0.41 × 0.36 × 0.14 mm

### Data collection  

Bruker APEXII CCD diffractometer
88935 measured reflections
11407 independent reflections
10339 reflections with *I* > 2σ(*I*)
*R*_int_ = 0.034

### Refinement  

*R*[*F* ^2^ > 2σ(*F* ^2^)] = 0.059
*wR*(*F* ^2^) = 0.122
*S* = 1.19
11407 reflections
485 parameters
18 restraints
H-atom parameters constrained
Δρ_max_ = 0.38 e Å^−3^
Δρ_min_ = −0.42 e Å^−3^

### 

Data collection: *APEX2* (Bruker, 2009 ▸); cell refinement: *SAINT* (Bruker, 2009 ▸); data reduction: *SAINT*; program(s) used to solve structure: *SHELXS97* (Sheldrick 2008 ▸); program(s) used to refine structure: *SHELXL2013* (Sheldrick, 2015 ▸); molecular graphics: *SHELXTL* (Sheldrick, 2008 ▸); software used to prepare material for publication: *SHELXTL* and *PLATON* (Spek, 2009 ▸).

## Supplementary Material

Crystal structure: contains datablock(s) I, global. DOI: 10.1107/S2056989015002273/sj5443sup1.cif

Structure factors: contains datablock(s) I. DOI: 10.1107/S2056989015002273/sj5443Isup2.hkl

Supporting information file. DOI: 10.1107/S2056989015002273/sj5443Isup3.cml

**Fig. 1.**
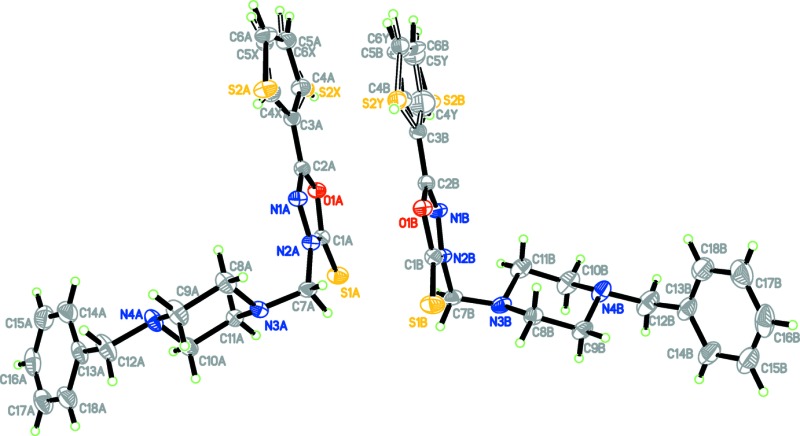
The molecular structure of the title compound with atom labels and 50% probability displacement ellipsoids (atoms of the minor part are labelled with the suffix X and Y for molecules A and B, respectively)

X Y A B . DOI: 10.1107/S2056989015002273/sj5443fig1.tif
The mol­ecular structure of the title compound with atom labels and 50% probability displacement ellipsoids (atoms of the minor part are labelled with the suffix *X* and *Y* for mol­ecules *A* and *B*, respectively)

**Fig. 2.**
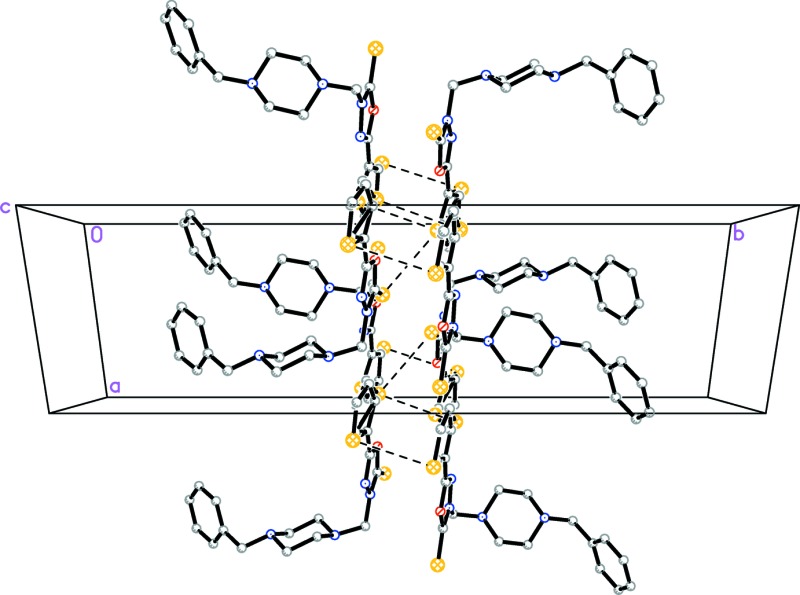
Crystal packing of the title compound, showing the S···S short contacts and π–π interactions as dashed lines. Only the major components of the thiophene rings are shown. All the H atoms are omitted for clarity.

. DOI: 10.1107/S2056989015002273/sj5443fig2.tif
Crystal packing of the title compound, showing the S⋯S short contacts and π–π inter­actions as dashed lines. Only the major components of the thio­phene rings are shown. All the H atoms are omitted for clarity.

CCDC reference: 1047059

Additional supporting information:  crystallographic information; 3D view; checkCIF report

## supplementary crystallographic information

### S1. Chemical context

Considerable attention has been devoted to 1,3,4-oxa­diazole derivatives which have long been known for their diverse chemotherapeutic properties as anti­viral agents against HIV-1 viruses (El-Emam *et al.*, 2004) and to demonstrate anti­bacterial (Padmavathi *et al.*, 2009) and anti-inflammatory properties (Kadi *et al.*, 2007; Al-Deeb *et al.*, 2006). The title compound was synthesized among a series of 2-thienyl-1,3,4-oxa­diazo­les and related derivatives as potential anti­microbial agents (Al-Omar, 2010).

### S2. Structural commentary

The asymmetric unit of the title compound consists of two crystallographically independent molecules (*A* and *B*) as shown in Fig. 1. The thio­phene rings has an approximately 180° rotational disorder (atoms of the minor part are labelled with the suffix *X* and *Y* for molecules *A* and *B*, respectively) about the C2–C3 single bond. The bond lengths and angles are within normal ranges and are comparable with those reported earlier for similar structures (El-Emam *et al.* 2012, 2013). The ratio of the refined site-occupancy factors of the major and minor parts of the disordered thio­phene ring is 0.8: 0.2 and 0.9 : 0.1 in molecules *A* and *B* respectively. The 1,3,4-oxa­diazole-2-thione (O1A/N1A/N2A/C1A/C2A and O1B/N1B/N2B/C1B/C2B) rings form dihedral angles of 7.71 (16), 10.0 (11) and 77.50 (12)° (molecule *A*); and 6.5 (3), 6.0 (9) and 55.30 (12)° (molecule *B*) with the major and minor parts of the disordered thio­phene ring and the mean plane of the adjacent piperazine rings (N3A/N4A/C8A–C11A and N3B/N4B/C8B–C11B), resulting in approximately V-shaped conformations for the molecules. The piperazine adopts a chair conformation with puckering parameters: Q = 0.591 (2) Å, θ = 178.50 (19)°, and φ = 8(10)° in molecule *A* and Q = 0.589 (2) Å, θ = 3.10 (19)°, and φ = 178 (6)° in molecule *B*. The terminal benzene rings (C13A–C18A and C13B–C18B) in both the molecules are inclined towards the mean plane of the piperazine ring with torsion angles N4A–C12A–C13A–C14A of -58.2 (3)° and N4B–C12B–C13B–C18B of -66.2 (3)°.

### S3. Supra­molecular features

In the crystal, no significant inter­molecular hydrogen bonds are observed. The crystal packing (Fig. 2) is stabilized by a short S···S contact [3.4792 (9) Å] and π–π inter­actions with *C*g1···*C*g2^i^ distance = 3.661 (3) Å, *C*g1···*C*g3^i^ distance = 3.664 (11) Å and *C*g4···*C*g5^i^ distance = 3.5727 (10) Å (symmetry code: (i) X, Y, Z), producing a three-dimensional structure. *C*g1–*C*g5 are the centroids of the S2A/C3A/C4A/C5A/C6A, S2B/C3B/C4B/C5B/C6B, C3B/S2Y/C6Y/C5Y/C4Y, O1A/C1A/N2A/N1A/C2A and O1B/C1B/N2B/N1B/C2B rings, respectively.

### S4. Synthesis and crystallization

The title compound was prepared by a literature procedure (Al-Omar, 2010) and crystallized from EtOH/CHCl_3_ (1:1) to yield colorless crystals. M. P.: 101–103°C.

### S5. Refinement details

All H atoms were positioned geometrically (C=H = 0.93 or 0.97 Å) and refined using a riding model with *U*_iso_(H) = 1.2 *U*_eq_(C). The thienyl ring is disordered over two positions and,in the final refinement cycles, the occupancy ratios were fixed in a ratio 0.8: 0.2 for molecule A and 0.9:0.1 for molecule *B*, respectively. Similarity and rigid-bond restraints were applied to the disordered atoms.

### Crystal data

**Table:**

C_18_H_20_N_4_OS_2_	*F*(000) = 1568
*M**_r_* = 372.50	*D*_x_ = 1.323 Mg m^−^^3^
Monoclinic, *P*2_1_/*c*	Mo *K*α radiation, λ = 0.71073 Å
*a* = 10.6909 (5) Å	Cell parameters from 9237 reflections
*b* = 29.3658 (13) Å	θ = 2.4–30.5°
*c* = 15.6179 (6) Å	µ = 0.30 mm^−^^1^
β = 130.283 (2)°	*T* = 273 K
*V* = 3740.4 (3) Å^3^	Plate, colourless
*Z* = 8	0.41 × 0.36 × 0.14 mm

### Data collection

**Table:**

Bruker APEXII CCD diffractometer	*R*_int_ = 0.034
φ and ω scans	θ_max_ = 30.6°, θ_min_ = 2.2°
88935 measured reflections	*h* = −15→15
11407 independent reflections	*k* = −42→41
10339 reflections with *I* > 2σ(*I*)	*l* = −22→22

### Refinement

**Table:**

Refinement on *F*^2^	18 restraints
Least-squares matrix: full	Hydrogen site location: inferred from neighbouring sites
*R*[*F*^2^ > 2σ(*F*^2^)] = 0.059	H-atom parameters constrained
*wR*(*F*^2^) = 0.122	*w* = 1/[σ^2^(*F*_o_^2^) + (0.0207*P*)^2^ + 3.9136*P*] where *P* = (*F*_o_^2^ + 2*F*_c_^2^)/3
*S* = 1.19	(Δ/σ)_max_ = 0.001
11407 reflections	Δρ_max_ = 0.38 e Å^−^^3^
485 parameters	Δρ_min_ = −0.42 e Å^−^^3^

### Special details

**Table:**

Geometry. All e.s.d.'s (except the e.s.d. in the dihedral angle between two l.s. planes) are estimated using the full covariance matrix. The cell e.s.d.'s are taken into account individually in the estimation of e.s.d.'s in distances, angles and torsion angles; correlations between e.s.d.'s in cell parameters are only used when they are defined by crystal symmetry. An approximate (isotropic) treatment of cell e.s.d.'s is used for estimating e.s.d.'s involving l.s. planes.

### Fractional atomic coordinates and isotropic or equivalent isotropic displacement parameters (Å^2^)

**Table:**

	*x*	*y*	*z*	*U*_iso_*/*U*_eq_	Occ. (<1)
S1B	0.86597 (6)	0.54555 (2)	0.95382 (4)	0.03328 (11)	
O1B	0.56261 (15)	0.54910 (4)	0.88376 (10)	0.0241 (2)	
N1B	0.40511 (17)	0.56647 (5)	0.70234 (13)	0.0242 (3)	
N2B	0.56914 (17)	0.56405 (5)	0.75134 (13)	0.0240 (3)	
N3B	0.64150 (18)	0.61694 (6)	0.66687 (13)	0.0260 (3)	
N4B	0.64546 (19)	0.71415 (6)	0.67316 (13)	0.0310 (3)	
C1B	0.6665 (2)	0.55360 (6)	0.86079 (15)	0.0241 (3)	
C2B	0.4084 (2)	0.55740 (6)	0.78476 (14)	0.0219 (3)	
C3B	0.2721 (2)	0.55573 (6)	0.78285 (15)	0.0225 (3)	
C4B	0.2698 (4)	0.54323 (10)	0.8668 (3)	0.0270 (6)	0.8
H4BA	0.3622	0.5338	0.9377	0.032*	0.8
C5B	0.1129 (8)	0.5463 (4)	0.8336 (6)	0.0365 (16)	0.8
H5BA	0.0886	0.5386	0.8793	0.044*	0.8
C6B	0.0010 (6)	0.5619 (3)	0.7273 (5)	0.0358 (13)	0.8
H6BA	−0.1088	0.5666	0.6922	0.043*	0.8
S2B	0.08174 (8)	0.57204 (3)	0.66379 (7)	0.03174 (14)	0.8
C4Y	0.122 (2)	0.5646 (6)	0.7033 (14)	0.055 (5)*	0.2
H4YA	0.0848	0.5722	0.6324	0.066*	0.2
C5Y	0.015 (3)	0.5625 (12)	0.725 (2)	0.039 (6)*	0.2
H5YA	−0.0966	0.5694	0.6747	0.046*	0.2
C6Y	0.103 (2)	0.5484 (12)	0.835 (2)	0.023 (4)*	0.2
H6YA	0.0573	0.5449	0.8691	0.028*	0.2
S2Y	0.3049 (4)	0.53795 (12)	0.9013 (3)	0.0243 (7)*	0.2
C7B	0.6198 (2)	0.57087 (7)	0.68367 (16)	0.0270 (4)	
H7BA	0.7222	0.5548	0.7199	0.032*	
H7BB	0.5381	0.5570	0.6107	0.032*	
C8B	0.7695 (2)	0.64187 (6)	0.77012 (14)	0.0256 (3)	
H8BA	0.8706	0.6245	0.8143	0.031*	
H8BB	0.7364	0.6461	0.8146	0.031*	
C9B	0.7969 (2)	0.68769 (7)	0.74051 (16)	0.0302 (4)	
H9BA	0.8811	0.7042	0.8087	0.036*	
H9BB	0.8345	0.6833	0.6988	0.036*	
C10B	0.5211 (3)	0.68905 (8)	0.56929 (17)	0.0386 (5)	
H10C	0.5586	0.6848	0.5275	0.046*	
H10D	0.4201	0.7065	0.5230	0.046*	
C11B	0.4893 (2)	0.64320 (7)	0.59601 (16)	0.0317 (4)	
H11C	0.4467	0.6474	0.6346	0.038*	
H11D	0.4079	0.6267	0.5269	0.038*	
C12B	0.6695 (3)	0.75944 (8)	0.64694 (19)	0.0439 (6)	
H12C	0.5643	0.7747	0.5963	0.053*	
H12D	0.7130	0.7563	0.6087	0.053*	
C13B	0.7846 (2)	0.78830 (7)	0.75006 (19)	0.0365 (5)	
C14B	0.9356 (3)	0.80127 (8)	0.7843 (2)	0.0442 (5)	
H14B	0.9656	0.7920	0.7428	0.053*	
C15B	1.0420 (3)	0.82762 (8)	0.8787 (2)	0.0504 (7)	
H15B	1.1426	0.8361	0.9002	0.061*	
C16B	1.0000 (3)	0.84134 (8)	0.9410 (2)	0.0536 (7)	
H16B	1.0720	0.8591	1.0048	0.064*	
C17B	0.8495 (3)	0.82871 (8)	0.9084 (3)	0.0533 (6)	
H17B	0.8205	0.8379	0.9505	0.064*	
C18B	0.7424 (3)	0.80236 (8)	0.8128 (2)	0.0458 (6)	
H18B	0.6413	0.7941	0.7909	0.055*	
S1A	0.40017 (6)	0.46380 (2)	0.54540 (4)	0.02973 (11)	
O1A	0.22909 (14)	0.45426 (4)	0.61460 (10)	0.0228 (2)	
N1A	0.41622 (17)	0.43676 (5)	0.79594 (12)	0.0224 (3)	
N2A	0.48826 (17)	0.44323 (5)	0.74796 (12)	0.0212 (3)	
N3A	0.71546 (17)	0.38885 (5)	0.83070 (12)	0.0216 (3)	
N4A	0.7102 (2)	0.29173 (5)	0.83187 (13)	0.0288 (3)	
C1A	0.3773 (2)	0.45354 (6)	0.63837 (14)	0.0218 (3)	
C2A	0.2620 (2)	0.44351 (6)	0.71288 (14)	0.0207 (3)	
C3A	0.1288 (2)	0.44023 (6)	0.71368 (14)	0.0218 (3)	
C4A	−0.0338 (3)	0.45153 (8)	0.6297 (2)	0.0265 (5)	0.9
H4AA	−0.0761	0.4630	0.5601	0.032*	0.9
C5A	−0.1282 (5)	0.4435 (2)	0.6622 (4)	0.0308 (11)	0.9
H5AA	−0.2400	0.4494	0.6163	0.037*	0.9
C6A	−0.0374 (5)	0.4264 (2)	0.7679 (4)	0.0303 (9)	0.9
H6AA	−0.0808	0.4187	0.8018	0.036*	0.9
S2A	0.16500 (7)	0.42031 (2)	0.83162 (5)	0.02979 (12)	0.9
C4X	0.125 (2)	0.4241 (7)	0.7841 (18)	0.030 (4)*	0.1
H4XA	0.2194	0.4120	0.8506	0.036*	0.1
C5X	−0.025 (4)	0.424 (2)	0.762 (3)	0.022 (6)*	0.1
H5XA	−0.0411	0.4155	0.8118	0.027*	0.1
C6X	−0.145 (4)	0.4397 (16)	0.654 (3)	0.010 (5)*	0.1
H6XA	−0.2563	0.4407	0.6182	0.012*	0.1
S2X	−0.0673 (7)	0.45671 (18)	0.5915 (4)	0.0191 (11)*	0.1
C7A	0.6656 (2)	0.43542 (6)	0.81358 (15)	0.0229 (3)	
H7AA	0.6985	0.4506	0.7757	0.028*	
H7AB	0.7244	0.4497	0.8864	0.028*	
C8A	0.7067 (3)	0.36412 (7)	0.90800 (16)	0.0296 (4)	
H8AA	0.5930	0.3599	0.8741	0.035*	
H8AB	0.7609	0.3815	0.9766	0.035*	
C9A	0.7893 (3)	0.31819 (7)	0.93468 (16)	0.0351 (4)	
H9AA	0.9042	0.3225	0.9717	0.042*	
H9AB	0.7828	0.3016	0.9854	0.042*	
C10A	0.7223 (2)	0.31688 (6)	0.75678 (15)	0.0267 (4)	
H10A	0.6714	0.2994	0.6887	0.032*	
H10B	0.8368	0.3213	0.7929	0.032*	
C11A	0.6385 (2)	0.36268 (6)	0.72752 (14)	0.0218 (3)	
H11A	0.6480	0.3792	0.6782	0.026*	
H11B	0.5230	0.3584	0.6887	0.026*	
C12A	0.7871 (3)	0.24680 (7)	0.85974 (18)	0.0402 (5)	
H12A	0.7855	0.2325	0.9150	0.048*	
H12B	0.9008	0.2506	0.8933	0.048*	
C13A	0.7034 (3)	0.21574 (6)	0.75928 (17)	0.0330 (4)	
C14A	0.5388 (3)	0.20464 (8)	0.6944 (2)	0.0407 (5)	
H14A	0.4774	0.2169	0.7117	0.049*	
C15A	0.4650 (3)	0.17530 (8)	0.6036 (2)	0.0450 (5)	
H15A	0.3545	0.1681	0.5605	0.054*	
C16A	0.5548 (3)	0.15663 (7)	0.5769 (2)	0.0433 (5)	
H16A	0.5049	0.1372	0.5157	0.052*	
C17A	0.7186 (3)	0.16709 (7)	0.6416 (2)	0.0433 (5)	
H17A	0.7800	0.1543	0.6248	0.052*	
C18A	0.7922 (3)	0.19659 (7)	0.7316 (2)	0.0382 (5)	
H18A	0.9027	0.2037	0.7742	0.046*	

### Atomic displacement parameters (Å^2^)

**Table:**

	*U*^11^	*U*^22^	*U*^33^	*U*^12^	*U*^13^	*U*^23^
S1B	0.0208 (2)	0.0416 (3)	0.0332 (2)	0.00472 (18)	0.01552 (19)	0.0035 (2)
O1B	0.0221 (6)	0.0267 (6)	0.0252 (6)	−0.0003 (5)	0.0160 (5)	−0.0005 (5)
N1B	0.0194 (6)	0.0317 (8)	0.0249 (7)	−0.0051 (6)	0.0159 (6)	−0.0065 (6)
N2B	0.0185 (6)	0.0310 (8)	0.0250 (7)	−0.0034 (5)	0.0152 (6)	−0.0047 (6)
N3B	0.0204 (7)	0.0353 (8)	0.0221 (7)	−0.0060 (6)	0.0137 (6)	−0.0054 (6)
N4B	0.0224 (7)	0.0316 (8)	0.0254 (8)	−0.0034 (6)	0.0094 (6)	0.0073 (6)
C1B	0.0237 (8)	0.0220 (8)	0.0295 (8)	−0.0013 (6)	0.0186 (7)	−0.0034 (6)
C2B	0.0206 (7)	0.0219 (8)	0.0246 (8)	−0.0025 (6)	0.0153 (7)	−0.0045 (6)
C3B	0.0225 (7)	0.0228 (8)	0.0261 (8)	−0.0037 (6)	0.0174 (7)	−0.0054 (6)
C4B	0.0241 (13)	0.0294 (13)	0.0281 (15)	−0.0001 (10)	0.0171 (13)	−0.0019 (12)
C5B	0.041 (2)	0.042 (3)	0.046 (2)	−0.0133 (15)	0.0372 (19)	−0.0147 (13)
C6B	0.0233 (15)	0.056 (2)	0.0382 (18)	−0.0136 (13)	0.0243 (15)	−0.0155 (13)
S2B	0.0208 (3)	0.0468 (4)	0.0288 (3)	−0.0045 (3)	0.0166 (3)	−0.0058 (3)
C7B	0.0259 (8)	0.0342 (9)	0.0297 (9)	−0.0057 (7)	0.0219 (7)	−0.0085 (7)
C8B	0.0182 (7)	0.0303 (9)	0.0216 (8)	−0.0015 (6)	0.0099 (7)	−0.0016 (7)
C9B	0.0192 (8)	0.0359 (10)	0.0273 (9)	−0.0051 (7)	0.0113 (7)	0.0011 (7)
C10B	0.0272 (9)	0.0465 (12)	0.0224 (9)	−0.0055 (8)	0.0072 (8)	0.0069 (8)
C11B	0.0198 (8)	0.0413 (11)	0.0220 (8)	−0.0054 (7)	0.0081 (7)	−0.0003 (7)
C12B	0.0359 (11)	0.0420 (12)	0.0372 (11)	−0.0041 (9)	0.0163 (10)	0.0159 (9)
C13B	0.0266 (9)	0.0269 (9)	0.0408 (11)	0.0001 (7)	0.0150 (9)	0.0139 (8)
C14B	0.0326 (10)	0.0374 (11)	0.0535 (14)	−0.0034 (9)	0.0238 (10)	0.0112 (10)
C15B	0.0289 (10)	0.0337 (11)	0.0622 (16)	−0.0023 (9)	0.0175 (11)	0.0104 (11)
C16B	0.0430 (13)	0.0246 (10)	0.0505 (14)	0.0019 (9)	0.0110 (11)	0.0058 (10)
C17B	0.0570 (15)	0.0327 (12)	0.0638 (17)	0.0086 (11)	0.0363 (14)	0.0064 (11)
C18B	0.0333 (11)	0.0344 (11)	0.0604 (15)	0.0036 (9)	0.0261 (11)	0.0099 (10)
S1A	0.0307 (2)	0.0384 (3)	0.0258 (2)	0.00467 (19)	0.02082 (19)	0.00510 (18)
O1A	0.0198 (5)	0.0271 (6)	0.0211 (6)	0.0010 (5)	0.0130 (5)	0.0004 (5)
N1A	0.0204 (6)	0.0269 (7)	0.0221 (7)	−0.0001 (5)	0.0147 (6)	−0.0013 (5)
N2A	0.0195 (6)	0.0243 (7)	0.0216 (6)	0.0011 (5)	0.0141 (6)	−0.0001 (5)
N3A	0.0192 (6)	0.0242 (7)	0.0200 (6)	0.0013 (5)	0.0120 (6)	−0.0014 (5)
N4A	0.0396 (9)	0.0247 (7)	0.0245 (7)	0.0114 (6)	0.0218 (7)	0.0063 (6)
C1A	0.0209 (7)	0.0202 (7)	0.0245 (8)	0.0012 (6)	0.0148 (7)	−0.0002 (6)
C2A	0.0217 (7)	0.0200 (7)	0.0217 (7)	−0.0002 (6)	0.0146 (6)	−0.0020 (6)
C3A	0.0194 (7)	0.0225 (8)	0.0230 (8)	−0.0011 (6)	0.0135 (6)	−0.0024 (6)
C4A	0.0231 (10)	0.0308 (11)	0.0251 (12)	−0.0005 (8)	0.0154 (10)	−0.0001 (10)
C5A	0.0218 (14)	0.033 (2)	0.0358 (17)	−0.0034 (13)	0.0179 (13)	−0.0032 (13)
C6A	0.0215 (12)	0.0390 (17)	0.0345 (14)	−0.0028 (10)	0.0200 (11)	−0.0025 (11)
S2A	0.0218 (2)	0.0423 (3)	0.0259 (3)	−0.0006 (2)	0.0158 (2)	0.0029 (2)
C7A	0.0170 (7)	0.0260 (8)	0.0239 (8)	−0.0023 (6)	0.0123 (6)	−0.0037 (6)
C8A	0.0387 (10)	0.0305 (9)	0.0233 (8)	0.0077 (8)	0.0218 (8)	0.0032 (7)
C9A	0.0461 (12)	0.0333 (10)	0.0218 (8)	0.0142 (9)	0.0201 (9)	0.0061 (7)
C10A	0.0304 (9)	0.0285 (9)	0.0244 (8)	0.0067 (7)	0.0191 (7)	0.0019 (7)
C11A	0.0228 (7)	0.0235 (8)	0.0185 (7)	0.0012 (6)	0.0130 (6)	0.0006 (6)
C12A	0.0545 (13)	0.0310 (10)	0.0327 (10)	0.0205 (9)	0.0272 (10)	0.0101 (8)
C13A	0.0525 (12)	0.0221 (8)	0.0351 (10)	0.0144 (8)	0.0331 (10)	0.0109 (7)
C14A	0.0559 (14)	0.0341 (11)	0.0505 (13)	0.0101 (10)	0.0427 (12)	0.0072 (9)
C15A	0.0579 (15)	0.0338 (11)	0.0504 (14)	0.0004 (10)	0.0382 (13)	0.0051 (10)
C16A	0.0722 (17)	0.0240 (9)	0.0413 (12)	0.0055 (10)	0.0402 (12)	0.0065 (8)
C17A	0.0719 (16)	0.0300 (10)	0.0500 (13)	0.0129 (10)	0.0493 (13)	0.0064 (9)
C18A	0.0518 (13)	0.0305 (10)	0.0456 (12)	0.0117 (9)	0.0374 (11)	0.0063 (9)

### Geometric parameters (Å, º)

**Table:**

S1B—C1B	1.6452 (18)	S1A—C1A	1.6484 (18)
O1B—C2B	1.368 (2)	O1A—C2A	1.372 (2)
O1B—C1B	1.377 (2)	O1A—C1A	1.374 (2)
N1B—C2B	1.293 (2)	N1A—C2A	1.294 (2)
N1B—N2B	1.3894 (19)	N1A—N2A	1.3917 (19)
N2B—C1B	1.341 (2)	N2A—C1A	1.343 (2)
N2B—C7B	1.481 (2)	N2A—C7A	1.478 (2)
N3B—C7B	1.425 (2)	N3A—C7A	1.429 (2)
N3B—C11B	1.463 (2)	N3A—C8A	1.463 (2)
N3B—C8B	1.468 (2)	N3A—C11A	1.466 (2)
N4B—C9B	1.459 (2)	N4A—C10A	1.461 (2)
N4B—C12B	1.463 (3)	N4A—C9A	1.463 (2)
N4B—C10B	1.467 (3)	N4A—C12A	1.464 (2)
C2B—C3B	1.439 (2)	C2A—C3A	1.436 (2)
C3B—C4Y	1.267 (17)	C3A—C4X	1.22 (2)
C3B—C4B	1.377 (4)	C3A—C4A	1.380 (3)
C3B—S2B	1.7132 (19)	C3A—S2A	1.7213 (18)
C3B—S2Y	1.726 (4)	C3A—S2X	1.759 (6)
C4B—C5B	1.403 (6)	C4A—C5A	1.411 (4)
C4B—H4BA	0.9300	C4A—H4AA	0.9300
C5B—C6B	1.353 (5)	C5A—C6A	1.360 (4)
C5B—H5BA	0.9300	C5A—H5AA	0.9300
C6B—S2B	1.710 (5)	C6A—S2A	1.709 (4)
C6B—H6BA	0.9300	C6A—H6AA	0.9300
C4Y—C5Y	1.395 (18)	C4X—C5X	1.413 (19)
C4Y—H4YA	0.9300	C4X—H4XA	0.9300
C5Y—C6Y	1.384 (15)	C5X—C6X	1.383 (17)
C5Y—H5YA	0.9300	C5X—H5XA	0.9300
C6Y—S2Y	1.716 (18)	C6X—S2X	1.712 (19)
C6Y—H6YA	0.9300	C6X—H6XA	0.9300
C7B—H7BA	0.9700	C7A—H7AA	0.9700
C7B—H7BB	0.9700	C7A—H7AB	0.9700
C8B—C9B	1.512 (3)	C8A—C9A	1.515 (3)
C8B—H8BA	0.9700	C8A—H8AA	0.9700
C8B—H8BB	0.9700	C8A—H8AB	0.9700
C9B—H9BA	0.9700	C9A—H9AA	0.9700
C9B—H9BB	0.9700	C9A—H9AB	0.9700
C10B—C11B	1.512 (3)	C10A—C11A	1.513 (2)
C10B—H10C	0.9700	C10A—H10A	0.9700
C10B—H10D	0.9700	C10A—H10B	0.9700
C11B—H11C	0.9700	C11A—H11A	0.9700
C11B—H11D	0.9700	C11A—H11B	0.9700
C12B—C13B	1.504 (3)	C12A—C13A	1.510 (3)
C12B—H12C	0.9700	C12A—H12A	0.9700
C12B—H12D	0.9700	C12A—H12B	0.9700
C13B—C18B	1.381 (4)	C13A—C14A	1.387 (3)
C13B—C14B	1.386 (3)	C13A—C18A	1.391 (3)
C14B—C15B	1.378 (4)	C14A—C15A	1.388 (3)
C14B—H14B	0.9300	C14A—H14A	0.9300
C15B—C16B	1.369 (4)	C15A—C16A	1.384 (3)
C15B—H15B	0.9300	C15A—H15A	0.9300
C16B—C17B	1.389 (4)	C16A—C17A	1.375 (4)
C16B—H16B	0.9300	C16A—H16A	0.9300
C17B—C18B	1.388 (4)	C17A—C18A	1.384 (3)
C17B—H17B	0.9300	C17A—H17A	0.9300
C18B—H18B	0.9300	C18A—H18A	0.9300
			
C2B—O1B—C1B	105.92 (13)	C2A—O1A—C1A	106.11 (13)
C2B—N1B—N2B	103.24 (14)	C2A—N1A—N2A	103.26 (13)
C1B—N2B—N1B	112.16 (14)	C1A—N2A—N1A	112.15 (13)
C1B—N2B—C7B	126.86 (15)	C1A—N2A—C7A	126.78 (14)
N1B—N2B—C7B	120.94 (14)	N1A—N2A—C7A	120.91 (14)
C7B—N3B—C11B	113.95 (15)	C7A—N3A—C8A	114.47 (14)
C7B—N3B—C8B	114.72 (15)	C7A—N3A—C11A	114.82 (14)
C11B—N3B—C8B	111.03 (15)	C8A—N3A—C11A	110.91 (14)
C9B—N4B—C12B	111.82 (16)	C10A—N4A—C9A	108.92 (16)
C9B—N4B—C10B	108.63 (17)	C10A—N4A—C12A	111.72 (16)
C12B—N4B—C10B	110.18 (16)	C9A—N4A—C12A	109.72 (15)
N2B—C1B—O1B	105.16 (14)	N2A—C1A—O1A	105.17 (14)
N2B—C1B—S1B	130.80 (14)	N2A—C1A—S1A	130.72 (13)
O1B—C1B—S1B	124.01 (14)	O1A—C1A—S1A	124.11 (13)
N1B—C2B—O1B	113.52 (14)	N1A—C2A—O1A	113.30 (14)
N1B—C2B—C3B	127.69 (16)	N1A—C2A—C3A	127.53 (16)
O1B—C2B—C3B	118.79 (15)	O1A—C2A—C3A	119.16 (15)
C4Y—C3B—C2B	129.5 (7)	C4X—C3A—C2A	130.0 (9)
C4B—C3B—C2B	129.1 (2)	C4A—C3A—C2A	128.50 (18)
C4B—C3B—S2B	111.59 (17)	C4A—C3A—S2A	111.88 (15)
C2B—C3B—S2B	119.30 (13)	C2A—C3A—S2A	119.61 (13)
C4Y—C3B—S2Y	111.4 (7)	C4X—C3A—S2X	112.0 (10)
C2B—C3B—S2Y	119.13 (17)	C2A—C3A—S2X	117.7 (2)
C3B—C4B—C5B	112.6 (4)	C3A—C4A—C5A	111.8 (3)
C3B—C4B—H4BA	123.7	C3A—C4A—H4AA	124.1
C5B—C4B—H4BA	123.7	C5A—C4A—H4AA	124.1
C6B—C5B—C4B	111.8 (5)	C6A—C5A—C4A	112.5 (4)
C6B—C5B—H5BA	124.1	C6A—C5A—H5AA	123.7
C4B—C5B—H5BA	124.1	C4A—C5A—H5AA	123.7
C5B—C6B—S2B	113.2 (4)	C5A—C6A—S2A	112.7 (3)
C5B—C6B—H6BA	123.4	C5A—C6A—H6AA	123.6
S2B—C6B—H6BA	123.4	S2A—C6A—H6AA	123.6
C6B—S2B—C3B	90.74 (19)	C6A—S2A—C3A	90.99 (14)
C3B—C4Y—C5Y	118.1 (15)	C3A—C4X—C5X	118.3 (18)
C3B—C4Y—H4YA	120.9	C3A—C4X—H4XA	120.8
C5Y—C4Y—H4YA	120.9	C5X—C4X—H4XA	120.8
C6Y—C5Y—C4Y	108.0 (19)	C6X—C5X—C4X	108 (2)
C6Y—C5Y—H5YA	126.0	C6X—C5X—H5XA	126.1
C4Y—C5Y—H5YA	126.0	C4X—C5X—H5XA	126.1
C5Y—C6Y—S2Y	112.5 (17)	C5X—C6X—S2X	112.6 (19)
C5Y—C6Y—H6YA	123.7	C5X—C6X—H6XA	123.7
S2Y—C6Y—H6YA	123.7	S2X—C6X—H6XA	123.7
C6Y—S2Y—C3B	89.7 (8)	C6X—S2X—C3A	88.9 (10)
N3B—C7B—N2B	115.96 (15)	N3A—C7A—N2A	115.75 (14)
N3B—C7B—H7BA	108.3	N3A—C7A—H7AA	108.3
N2B—C7B—H7BA	108.3	N2A—C7A—H7AA	108.3
N3B—C7B—H7BB	108.3	N3A—C7A—H7AB	108.3
N2B—C7B—H7BB	108.3	N2A—C7A—H7AB	108.3
H7BA—C7B—H7BB	107.4	H7AA—C7A—H7AB	107.4
N3B—C8B—C9B	109.59 (15)	N3A—C8A—C9A	109.39 (15)
N3B—C8B—H8BA	109.8	N3A—C8A—H8AA	109.8
C9B—C8B—H8BA	109.8	C9A—C8A—H8AA	109.8
N3B—C8B—H8BB	109.8	N3A—C8A—H8AB	109.8
C9B—C8B—H8BB	109.8	C9A—C8A—H8AB	109.8
H8BA—C8B—H8BB	108.2	H8AA—C8A—H8AB	108.2
N4B—C9B—C8B	110.47 (15)	N4A—C9A—C8A	110.51 (15)
N4B—C9B—H9BA	109.6	N4A—C9A—H9AA	109.5
C8B—C9B—H9BA	109.6	C8A—C9A—H9AA	109.5
N4B—C9B—H9BB	109.6	N4A—C9A—H9AB	109.5
C8B—C9B—H9BB	109.6	C8A—C9A—H9AB	109.5
H9BA—C9B—H9BB	108.1	H9AA—C9A—H9AB	108.1
N4B—C10B—C11B	110.30 (16)	N4A—C10A—C11A	110.44 (14)
N4B—C10B—H10C	109.6	N4A—C10A—H10A	109.6
C11B—C10B—H10C	109.6	C11A—C10A—H10A	109.6
N4B—C10B—H10D	109.6	N4A—C10A—H10B	109.6
C11B—C10B—H10D	109.6	C11A—C10A—H10B	109.6
H10C—C10B—H10D	108.1	H10A—C10A—H10B	108.1
N3B—C11B—C10B	109.84 (16)	N3A—C11A—C10A	109.39 (14)
N3B—C11B—H11C	109.7	N3A—C11A—H11A	109.8
C10B—C11B—H11C	109.7	C10A—C11A—H11A	109.8
N3B—C11B—H11D	109.7	N3A—C11A—H11B	109.8
C10B—C11B—H11D	109.7	C10A—C11A—H11B	109.8
H11C—C11B—H11D	108.2	H11A—C11A—H11B	108.2
N4B—C12B—C13B	112.51 (17)	N4A—C12A—C13A	113.20 (17)
N4B—C12B—H12C	109.1	N4A—C12A—H12A	108.9
C13B—C12B—H12C	109.1	C13A—C12A—H12A	108.9
N4B—C12B—H12D	109.1	N4A—C12A—H12B	108.9
C13B—C12B—H12D	109.1	C13A—C12A—H12B	108.9
H12C—C12B—H12D	107.8	H12A—C12A—H12B	107.8
C18B—C13B—C14B	118.5 (2)	C14A—C13A—C18A	118.5 (2)
C18B—C13B—C12B	120.8 (2)	C14A—C13A—C12A	121.40 (19)
C14B—C13B—C12B	120.7 (2)	C18A—C13A—C12A	120.1 (2)
C15B—C14B—C13B	121.2 (3)	C13A—C14A—C15A	120.5 (2)
C15B—C14B—H14B	119.4	C13A—C14A—H14A	119.8
C13B—C14B—H14B	119.4	C15A—C14A—H14A	119.8
C16B—C15B—C14B	120.1 (2)	C16A—C15A—C14A	120.4 (3)
C16B—C15B—H15B	119.9	C16A—C15A—H15A	119.8
C14B—C15B—H15B	119.9	C14A—C15A—H15A	119.8
C15B—C16B—C17B	119.7 (3)	C17A—C16A—C15A	119.4 (2)
C15B—C16B—H16B	120.1	C17A—C16A—H16A	120.3
C17B—C16B—H16B	120.1	C15A—C16A—H16A	120.3
C18B—C17B—C16B	119.8 (3)	C16A—C17A—C18A	120.3 (2)
C18B—C17B—H17B	120.1	C16A—C17A—H17A	119.8
C16B—C17B—H17B	120.1	C18A—C17A—H17A	119.8
C13B—C18B—C17B	120.6 (2)	C17A—C18A—C13A	120.9 (2)
C13B—C18B—H18B	119.7	C17A—C18A—H18A	119.5
C17B—C18B—H18B	119.7	C13A—C18A—H18A	119.5
			
C2B—N1B—N2B—C1B	0.15 (19)	C2A—N1A—N2A—C1A	−0.09 (19)
C2B—N1B—N2B—C7B	177.93 (16)	C2A—N1A—N2A—C7A	−175.66 (15)
N1B—N2B—C1B—O1B	−0.04 (19)	N1A—N2A—C1A—O1A	0.61 (18)
C7B—N2B—C1B—O1B	−177.65 (16)	C7A—N2A—C1A—O1A	175.86 (15)
N1B—N2B—C1B—S1B	178.23 (14)	N1A—N2A—C1A—S1A	−179.50 (13)
C7B—N2B—C1B—S1B	0.6 (3)	C7A—N2A—C1A—S1A	−4.2 (3)
C2B—O1B—C1B—N2B	−0.08 (18)	C2A—O1A—C1A—N2A	−0.86 (17)
C2B—O1B—C1B—S1B	−178.50 (13)	C2A—O1A—C1A—S1A	179.24 (13)
N2B—N1B—C2B—O1B	−0.21 (19)	N2A—N1A—C2A—O1A	−0.50 (18)
N2B—N1B—C2B—C3B	179.12 (17)	N2A—N1A—C2A—C3A	178.47 (16)
C1B—O1B—C2B—N1B	0.19 (19)	C1A—O1A—C2A—N1A	0.88 (19)
C1B—O1B—C2B—C3B	−179.20 (15)	C1A—O1A—C2A—C3A	−178.17 (15)
N1B—C2B—C3B—C4Y	−3.6 (11)	N1A—C2A—C3A—C4X	−12.6 (14)
O1B—C2B—C3B—C4Y	175.7 (11)	O1A—C2A—C3A—C4X	166.3 (14)
N1B—C2B—C3B—C4B	175.1 (2)	N1A—C2A—C3A—C4A	173.0 (2)
O1B—C2B—C3B—C4B	−5.6 (3)	O1A—C2A—C3A—C4A	−8.1 (3)
N1B—C2B—C3B—S2B	−6.3 (3)	N1A—C2A—C3A—S2A	−6.5 (3)
O1B—C2B—C3B—S2B	172.95 (12)	O1A—C2A—C3A—S2A	172.39 (12)
N1B—C2B—C3B—S2Y	174.4 (2)	N1A—C2A—C3A—S2X	173.9 (2)
O1B—C2B—C3B—S2Y	−6.3 (3)	O1A—C2A—C3A—S2X	−7.2 (3)
C4Y—C3B—C4B—C5B	−1.6 (10)	C4X—C3A—C4A—C5A	4.5 (11)
C2B—C3B—C4B—C5B	179.5 (5)	C2A—C3A—C4A—C5A	−179.9 (3)
S2B—C3B—C4B—C5B	0.9 (5)	S2A—C3A—C4A—C5A	−0.4 (4)
S2Y—C3B—C4B—C5B	−176.9 (15)	S2X—C3A—C4A—C5A	175.8 (14)
C3B—C4B—C5B—C6B	−1.4 (10)	C3A—C4A—C5A—C6A	−0.4 (6)
C4B—C5B—C6B—S2B	1.3 (10)	C4A—C5A—C6A—S2A	1.0 (7)
C5B—C6B—S2B—C3B	−0.7 (7)	C5A—C6A—S2A—C3A	−1.0 (5)
C4Y—C3B—S2B—C6B	13 (5)	C4X—C3A—S2A—C6A	−24 (5)
C4B—C3B—S2B—C6B	−0.1 (3)	C4A—C3A—S2A—C6A	0.8 (3)
C2B—C3B—S2B—C6B	−178.9 (3)	C2A—C3A—S2A—C6A	−179.6 (3)
S2Y—C3B—S2B—C6B	0.3 (3)	S2X—C3A—S2A—C6A	−0.1 (3)
C4B—C3B—C4Y—C5Y	4 (2)	C4A—C3A—C4X—C5X	−3 (3)
C2B—C3B—C4Y—C5Y	−176.7 (18)	C2A—C3A—C4X—C5X	−178 (3)
S2B—C3B—C4Y—C5Y	−163 (6)	S2A—C3A—C4X—C5X	154 (7)
S2Y—C3B—C4Y—C5Y	5 (2)	S2X—C3A—C4X—C5X	−5 (4)
C3B—C4Y—C5Y—C6Y	−3 (4)	C3A—C4X—C5X—C6X	6 (6)
C4Y—C5Y—C6Y—S2Y	−1 (4)	C4X—C5X—C6X—S2X	−5 (6)
C5Y—C6Y—S2Y—C3B	3 (3)	C5X—C6X—S2X—C3A	2 (4)
C4Y—C3B—S2Y—C6Y	−4.6 (16)	C4X—C3A—S2X—C6X	1 (2)
C4B—C3B—S2Y—C6Y	0.3 (17)	C4A—C3A—S2X—C6X	−8 (2)
C2B—C3B—S2Y—C6Y	177.1 (13)	C2A—C3A—S2X—C6X	175.9 (17)
S2B—C3B—S2Y—C6Y	−2.1 (13)	S2A—C3A—S2X—C6X	−3.6 (17)
C11B—N3B—C7B—N2B	−68.0 (2)	C8A—N3A—C7A—N2A	−70.88 (19)
C8B—N3B—C7B—N2B	61.6 (2)	C11A—N3A—C7A—N2A	59.11 (19)
C1B—N2B—C7B—N3B	−98.4 (2)	C1A—N2A—C7A—N3A	−98.7 (2)
N1B—N2B—C7B—N3B	84.2 (2)	N1A—N2A—C7A—N3A	76.1 (2)
C7B—N3B—C8B—C9B	172.08 (15)	C7A—N3A—C8A—C9A	−170.33 (16)
C11B—N3B—C8B—C9B	−56.94 (19)	C11A—N3A—C8A—C9A	57.8 (2)
C12B—N4B—C9B—C8B	177.67 (18)	C10A—N4A—C9A—C8A	59.8 (2)
C10B—N4B—C9B—C8B	−60.5 (2)	C12A—N4A—C9A—C8A	−177.65 (19)
N3B—C8B—C9B—N4B	59.0 (2)	N3A—C8A—C9A—N4A	−58.7 (2)
C9B—N4B—C10B—C11B	60.3 (2)	C9A—N4A—C10A—C11A	−59.9 (2)
C12B—N4B—C10B—C11B	−176.93 (19)	C12A—N4A—C10A—C11A	178.73 (17)
C7B—N3B—C11B—C10B	−171.68 (16)	C7A—N3A—C11A—C10A	170.34 (14)
C8B—N3B—C11B—C10B	56.9 (2)	C8A—N3A—C11A—C10A	−57.95 (18)
N4B—C10B—C11B—N3B	−58.7 (2)	N4A—C10A—C11A—N3A	59.00 (19)
C9B—N4B—C12B—C13B	−64.3 (3)	C10A—N4A—C12A—C13A	−64.0 (2)
C10B—N4B—C12B—C13B	174.8 (2)	C9A—N4A—C12A—C13A	175.08 (19)
N4B—C12B—C13B—C18B	−66.2 (3)	N4A—C12A—C13A—C14A	−58.2 (3)
N4B—C12B—C13B—C14B	113.6 (2)	N4A—C12A—C13A—C18A	123.5 (2)
C18B—C13B—C14B—C15B	0.0 (3)	C18A—C13A—C14A—C15A	−0.4 (3)
C12B—C13B—C14B—C15B	−179.9 (2)	C12A—C13A—C14A—C15A	−178.7 (2)
C13B—C14B—C15B—C16B	0.3 (4)	C13A—C14A—C15A—C16A	0.2 (3)
C14B—C15B—C16B—C17B	−0.2 (4)	C14A—C15A—C16A—C17A	0.5 (3)
C15B—C16B—C17B—C18B	−0.2 (4)	C15A—C16A—C17A—C18A	−1.0 (3)
C14B—C13B—C18B—C17B	−0.3 (3)	C16A—C17A—C18A—C13A	0.8 (3)
C12B—C13B—C18B—C17B	179.5 (2)	C14A—C13A—C18A—C17A	−0.1 (3)
C16B—C17B—C18B—C13B	0.4 (4)	C12A—C13A—C18A—C17A	178.27 (19)
