# Supplementary figures and images for: Crystal structure of 3-[(4-benzyl­piperazin-1-yl)meth­yl]-5-(thio­phen-2-yl)-2,3-di­hydro-1,3,4-oxa­diazole-2-thione
Source: Acta Crystallogr E Crystallogr Commun. 2015 Feb 13;71(Pt 3):o175–6. doi: 10.1107/S2056989015002273 (PMC4350721; doi:10.1107/S2056989015002273)

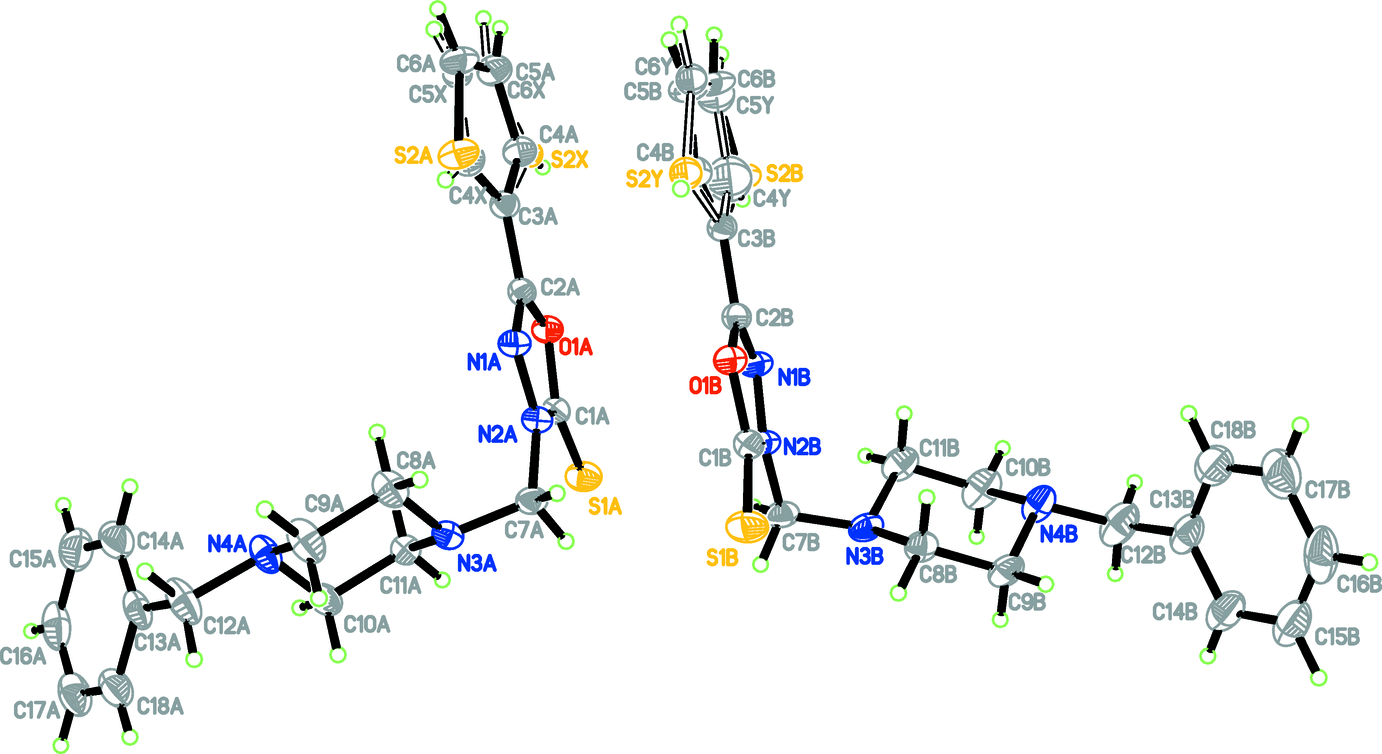

Supplement: Supplementary file 4 [file e-71-0o175-fig1.tif]

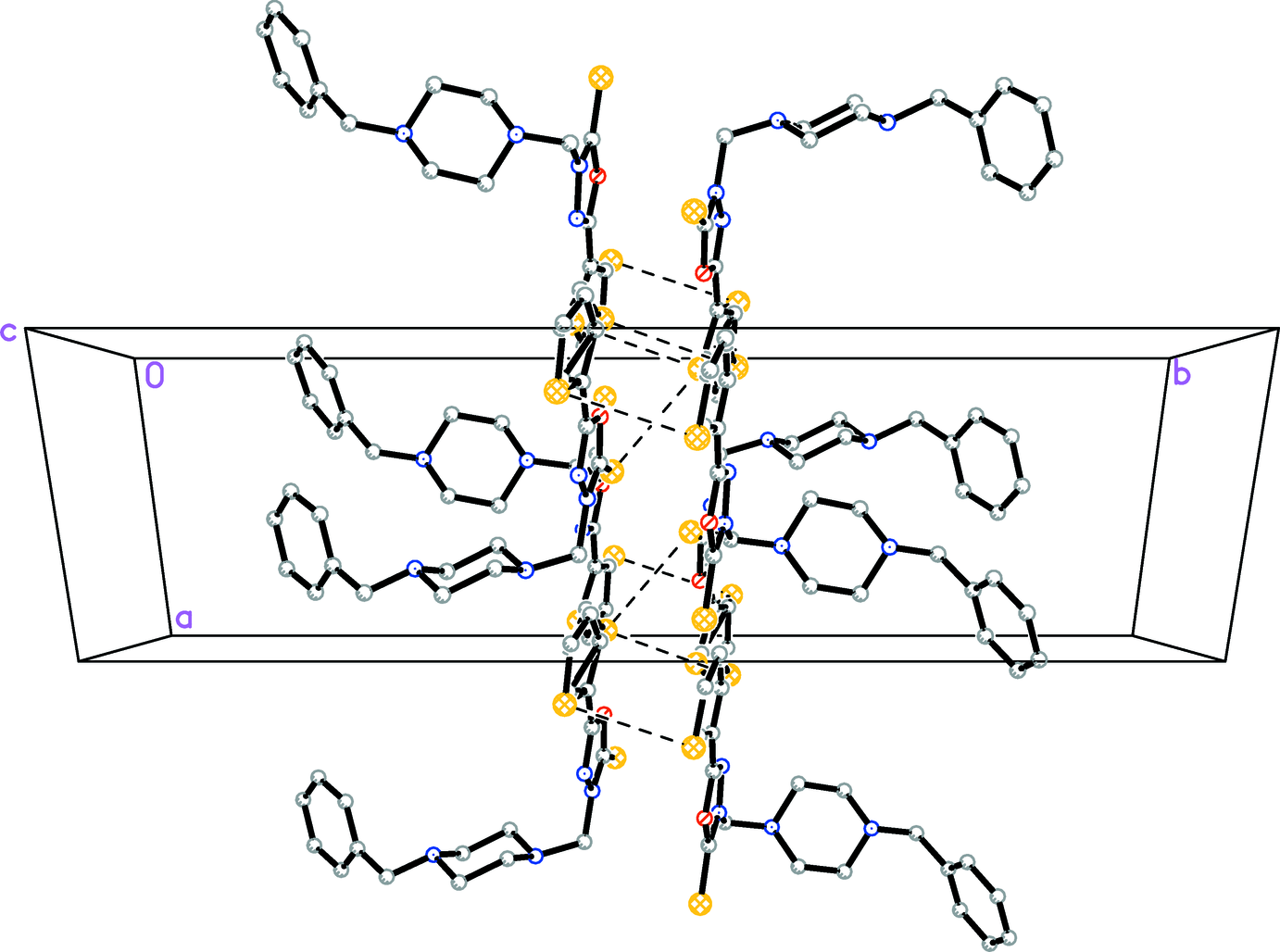

Supplement: Supplementary file 5 [file e-71-0o175-fig2.tif]
